# Effects of exercise‐induced beta‐hydroxybutyrate on muscle function and cognitive function

**DOI:** 10.14814/phy2.14497

**Published:** 2021-02-06

**Authors:** Seong Eun Kwak, Jun Hyun Bae, Ji Heun Lee, Hyung Eun Shin, DiDi Zhang, Sung Chun Cho, Wook Song

**Affiliations:** ^1^ Institute of Sport Science Seoul National University Seoul Korea; ^2^ Well Aging Research Center DGIST Daegu South Korea; ^3^ Institue on Aging Seoul National University Seoul Korea

**Keywords:** beta‐hydroxybutyrate, cognitive function, exercise, skeletal muscle

## Abstract

Recent studies have shown that exercise improves skeletal muscle and cognitive function by stimulating the secretion of numerous molecules. In particular, previous studies have suggested that exercise‐induced beta‐hydroxybutyrate (BHB) release might improve skeletal muscle and cognitive function, but to date these studies have been limited to cell and animal models. Therefore, we aimed to determine how an exercise‐induced increase in BHB affects skeletal muscle and cognitive function at a cellular level, in an animal model, and in humans. The effects of BHB on skeletal muscle and cognitive function were determined by treating C2C12 and C6 cell lines with BHB, and by measuring the skeletal muscle and serum BHB concentrations in aged mice after endurance or resistance exercise. In addition, serum BHB concentration was measured before and after high‐speed band exercise in elderly people, and its relationships with muscle and cognitive function were analyzed. We found that BHB increased cell viability and brain‐derived neurotrophic factor expression level in C6 cells, and endurance exercise, but not resistance exercise, increased the muscle BHB concentration in aged mice. Furthermore, the BHB concentration was positively related to skeletal muscle and cognitive function. Exercise did not increase the serum BHB concentration in the elderly people and BHB did not correlate with cognitive function, but after excluding the five people with the highest preexisting serum concentrations of BHB, the BHB concentrations of the remaining participants were increased by exercise, and the concentration showed a tendency toward a positive correlation with cognitive function. Thus, the BHB released by skeletal muscle following endurance exercise may improve muscle and cognitive function in animals and humans.

## Graphical abstract

Exercise upregulated DBHB level in skeletal muscle in mice and skeletal muscle cell. Also, DBHB effected to cognitive function as well.

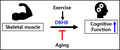

## NEW AND NOTEWORTHY

Our research on exercise‐induced “Beta‐hydroxybutyrate (BHB)” effects on skeletal muscle function and cognitive function is new and noteworthy. This research combines skeletal muscle function and cognitive function and analyze it with BHB induced by exercise. Especially, this study has never been performed through human sample, but in our research, we covered everything from cell study to human study. Therefore, we found that exercise‐induced BHB from skeletal muscle could improve muscle function and cognitive function. Therefore, our study is new and noteworthy.

## 1 INTRODUCTION

Elderly people frequently experience declines in skeletal muscle and cognitive function (Curcio et al., 2019; Dionyssiotis, 2019; Dudley‐Javoroski, Lee, & Shields, 2020; Sun, Lee, Yim, Won, & Ko, 2019; Yang, Jiang, Zeng, & Tang 2019; Zammit, Robitaille, Piccinin, Muniz‐Terrera, & Hofer, 2019). There is a great deal of evidence that skeletal muscle mass and function are related to cognitive function (Pedersen, 2019; Yoon, Lee, & Song, 2018). In addition, a number of mediators have been identified that are secreted by skeletal muscle, and are termed “myokines” (Islam, Young, & Wrann, 2017; Kwak, Cho, et al., 2019; Moon et al., 2016; Pedersen, 2019). Beta‐hydroxybutyrate is a ketone body that is released from the liver during starvation, when blood glucose concentrations are low, to maintain energy homeostasis (Dedkova & Blatter, 2014; Robinson & Williamson, 1980), and it is used by skeletal muscle to provide energy for its activity (Vice, Privette, Hickner, & Barakat, 2005). Beta‐hydroxybutyrate not only inhibits glycolysis and is used in the tricarboxylic acid cycle (Cahill & Aoki, 1980; Evans, Cogan, & Egan, 2017), but it also has effects on signaling pathways, including those involved in inflammation, lipolysis, metabolic rate, stress resistance, and longevity, such as via G‐protein‐coupled receptor (GPCR) binding (Rojas‐Morales, Tapia, & Pedraza‐Chaverri, 2016). Moreover, during exercise, the liver releases more BHB into the circulation, which inhibits histone deacetylases (HDACs) 2 and 3, which in turn are responsible for a reduction in the expression of brain‐derived neurotrophic factor (BDNF), a biomarker of cognitive function (Sleiman et al., 2016). Thus, BHB upregulates BDNF expression by inhibiting HDACs. Furthermore, in a previous study, BHB supplementation improved muscle and cognitive function in an animal model by improving mitochondrial function (Munroe et al., 2017) and reducing oxidative stress (Kong et al., 2017; Pinto, Bonucci, Maggi, Corsi, & Businaro, 2018; Wang et al., 2017). Thus, although BHB is a metabolite that was once recognized only as a by‐product of acetoacetate, it is now thought to be a mediator of cross‐talk between the brain and skeletal muscle (Pedersen, 2019). However, these previous studies were conducted in animal models or lines alone and only determined the effects of BHB supplementation on skeletal muscle and cognitive function (Munroe et al., 2017). Thus, the effects of BHB require confirmation in humans.

Previous studies showed that BHB is effective to skeletal muscle function and cognitive function in human and animal model. Eating BHB supplements upregulated skeletal muscle function and cognitive function in human. Also, many other studies showed effects of BHB on skeletal muscle function and cognitive function through animal models to human study (Evans et al., 2017; Munroe et al., 2017; Pinto et al., 2018; Vice et al., 2005). However, there are few studies related to exercise upregulated BHB level and resistance or endurance exercise.

In this study, we aimed to determine the effects of exercise on BHB concentrations in cells, animal model, and humans. From this study, it could be said that exercise upregulates BHB level from skeletal muscle and it improves cognitive function of the elderly. We show that BHB released by skeletal muscle during exercise has beneficial effects in muscle and especially on cognitive function.

## 2 MATERIALS AND METHODS

### 2.1 Animal care

The experiments were approved by the Institutional Animal Care and Use Committee of DIGIST (DGIST‐IACUC‐18050202‐00). C57Bl/6J male mice were housed in a facility that was maintained at 22°C, under a 12‐hr light/dark cycle, and had ad libitum access to food and water. The mice were allocated to groups according to their age and the type of exercise to be performed: a young group that did not exercise (YC, *n* = 4), an old group that did not exercise (OC, *n* = 3), an old group that performed endurance exercise on a treadmill (OEE, *n* = 4), and an old group that performed resistance exercise on a ladder (ORE, *n* = 3). The young group was 7 months old and the old groups were 27 months old.

### 2.2 Exercise protocol

Mice that performed treadmill exercise on a treadmill (Columbus, Exer 3/6) with a 10° incline, starting at 8 m/min for 30 min, 1–2 times per week, and the speed was increased by 2 m/min every 2 weeks. Ladder exercise was performed 3 days per week for 4 weeks, with the ladder being 1 m long, having a 1.5 interval grid, and being inclined at 85°. The ORE group started with 10% of its body mass. The ORE group climbed the ladder 10 times per session and the intensity of exercise was increased after four successful trials (Kwak, Cho, et al., 2019).

### 2.3 Grip strength

Forelimb grip strength was measured using a Grip Strength Meter (Bioseb) after finishing the 4 weeks of exercise. The mice were encouraged to grasp a grid plate attached to the force gauge, and gently pulled away from the gauge. Three measurements were made, the mean grip strength was calculated, and this was normalized to body mass (Kwak, Cho, et al., 2019).

### 2.4 Tissue collection

To avoid a residual effect of the last bout of exercise, terminal anesthesia was induced 48 hr after the final bout by intraperitoneal injection of 20% urethane solution, blood was collected by enucleation, and limb skeletal muscles were collected.

### 2.5 Real‐time PCR

RNA was extracted using TRIzol reagent (Invitrogen, #15596026) and reverse transcribed using CycleScript RT premix (Bioneer, #K‐2044‐CFG). The expression of BDNF was measured by real‐time PCR using SYBR Green with low ROX (Enzynomics, #RT500M) and a BioRad #CFX96 cycler. cDNA was amplified using 40 cycles, consisting of denaturation at 95°C for 10 s, annealing at 60°C for 15 s, and elongation at 72°C for 25 s. The primer sequences were: BDNF forward: 5‐TGGCCTAACAGTGTTTGCAG‐3, BDNF reverse 5‐GGATTTGAGTGTGGTTCTCC‐3, 18S forward 5‐GTAACCCGTTGAACCCCATT‐3, and 18S reverse 5‐CCATCCAATCGGTAGTAGCG‐3.

### 2.6 Isometric force test

*Soleus* (SOL) muscles were isolated from control and exercised mice to determine their contractile properties. The isolated muscles were put into a chamber containing 25°C Krebs–Ringer buffer (118 mM NaCl, 4.75 mM KCl, 24.8 mM NaHCO_3_, 1.18 mM KH_2_PO_4_, 2.5 mM CaCl_2_, 1.18 mM MgSO_4_, and 10 mM glucose, pH 7.4), through which 95% O_2_/5% CO_2_ was passed. The proximal tendon was fixed using a clamp and the distal tendon was fixed to a force transducer (Grass Instruments, FT.03). The optimum muscle length was determined as the length producing the highest twitch force (mN) following a supramaximal pulse of 0.2 ms duration. Isometric tetanic contractions (mN) were measured using an electrical stimulator (Grass Instruments, S48) at a frequency of 150 Hz for the SOL and 100 Hz for the *extensor digitorum longus* (EDL) muscle, a stimulation current of 100 V, and a duration of 800 ms (SOL) or 200 ms (EDL).

### 2.7 Measurement of BHB concentration

Serum and skeletal muscle BHB concentrations were measured using a BHB Assay kit (Cayman, #700190). Serum was diluted 2.5‐fold and skeletal muscles were first homogenized in the kit assay buffer.

### 2.8 Y‐Maze test

The cognitive function of the mice was assessed using a Y‐maze test, as performed previously (Kwak, Cho, et al., 2019). The Y‐maze had three arms that were each 60 cm long, 11.5 cm wide, and 25 cm high, and the angle between the arms was 120°. After 4 weeks of exercise, mice were placed in the Y‐maze and their movements across the arms were counted during 8‐min periods, with the three arms being labeled 1, 2, and 3. If a mouse did not pass the same number during the three movements, one point was awarded. The alteration percentage for each mouse was calculated using the total number of movements and the score (Kwak, Cho, et al., 2019).

### 2.9 In vitro study

Skeletal muscle C2C12 cells (ATCC, CRL‐1772) and brain glial C6 cells (KCLB, #10107, Korean cell line bank) were used. The cells were treated with BHB (Sigma, #54965‐10G‐F) at various concentrations. The C2C12 and C6 cells were treated with 0.5 µM doxorubicin to induce senescence and functional impairment (Bielak‐Zmijewska, 2014). To mimic exercise in the C2C12 cells, forskolin (Sigma–Aldrich, #F6886) was added to the culture medium for 24 hr, the cells were washed twice with 1 × PBS (Lonza, #17‐516F), and then Dulbecco's‐modified Eagle's Medium (DMEM, Welgene) was added for 4 hr. The medium was then collected and syringe‐filtered (Sartorius, #16534) to remove the cell debris, and used to treat C6 cells in 96‐well plates and 12‐well plates. The viability of these cells was assessed using a cell counting kit (Dojindo, #CK04) and BDNF mRNA expression was measured (Harris et al., 2004).

### 2.10 Collection of human serum and exercise intervention protocol

Elderly volunteers performed high‐speed elastic band training 3 days a week for 1 hr, as previously described (Yoon et al., 2018). During each session, the participants performed a 10‐min warm‐up, followed by 40 min of high‐speed elastic band training such as seated rowing, one leg press, applied pec deck flus, seated leg raise, lateral raise, semi‐squats, wide squats, and bridging using an elastic band, and 10 min of cooling down. There was at least 48 hr between sessions, and the exercise was performed under the direct supervision of an instructor, to make sure it was safe and the participants were accustomed to the exercise protocol. The exercise group used low‐tension elastic bands (20 Nm), and their rate of perceived exertion was maintained between 12 and 13 during exercise. The demographic data of participants are shown (Table 1). The protocol was approved by the institutional review board of Seoul National University.

**TABLE 1:** Demographics of human study

	Pre‐exercise	Post‐Exercise
Demographics		
Age, mean (*SD*)	71.79 ± 4.36	71.79 ± 4.36
Female, *n* (%)	17 (70.83%)	17 (70.83%)
MMSE, mean (*SD*)	24.83 ± 4.07	28.04 ± 2.84
Flexor, mean (*SD*)	14.97 ± 5.64	18.28 ± 4.67

### 2.11 Statistical analysis

Data are expressed as means ± SEMs. Statistical analysis was performed using Student's *t* test (one‐tailed or two‐tailed, unpaired). Relationships between two variables were analyzed using Spearman or Pearson's correlations.

## 3 RESULTS

### 3.1 The effects of aging and 4 weeks of exercise on muscle and serum BHB concentrations

**FIGURE 1:**
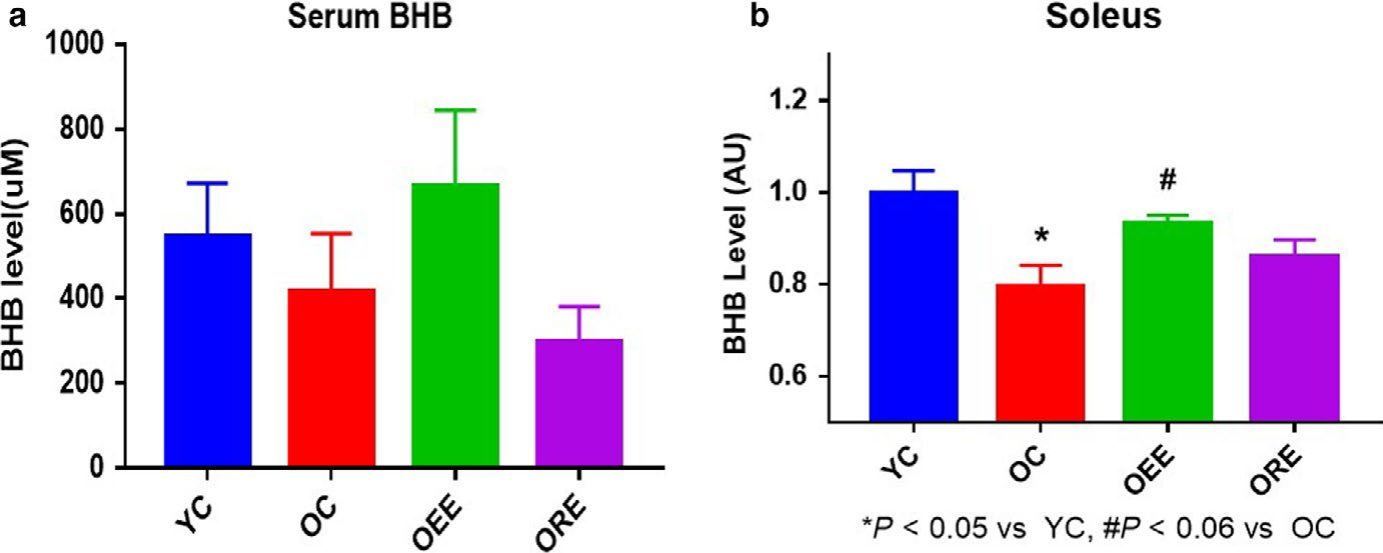
Effects of Exercise and Aging on Muscle and Serum beta‐hydroxybutyrate (BHB) Concentrations. (a and b) BHB concentrations in serum and soleus muscle in each animal group *p < .05 versus YC, ^#^ *p* < .05 versus OC. Statistical analysis was performed using two‐tailed Student's *t* tests. Data are means ± SEMs

**FIGURE 2:**
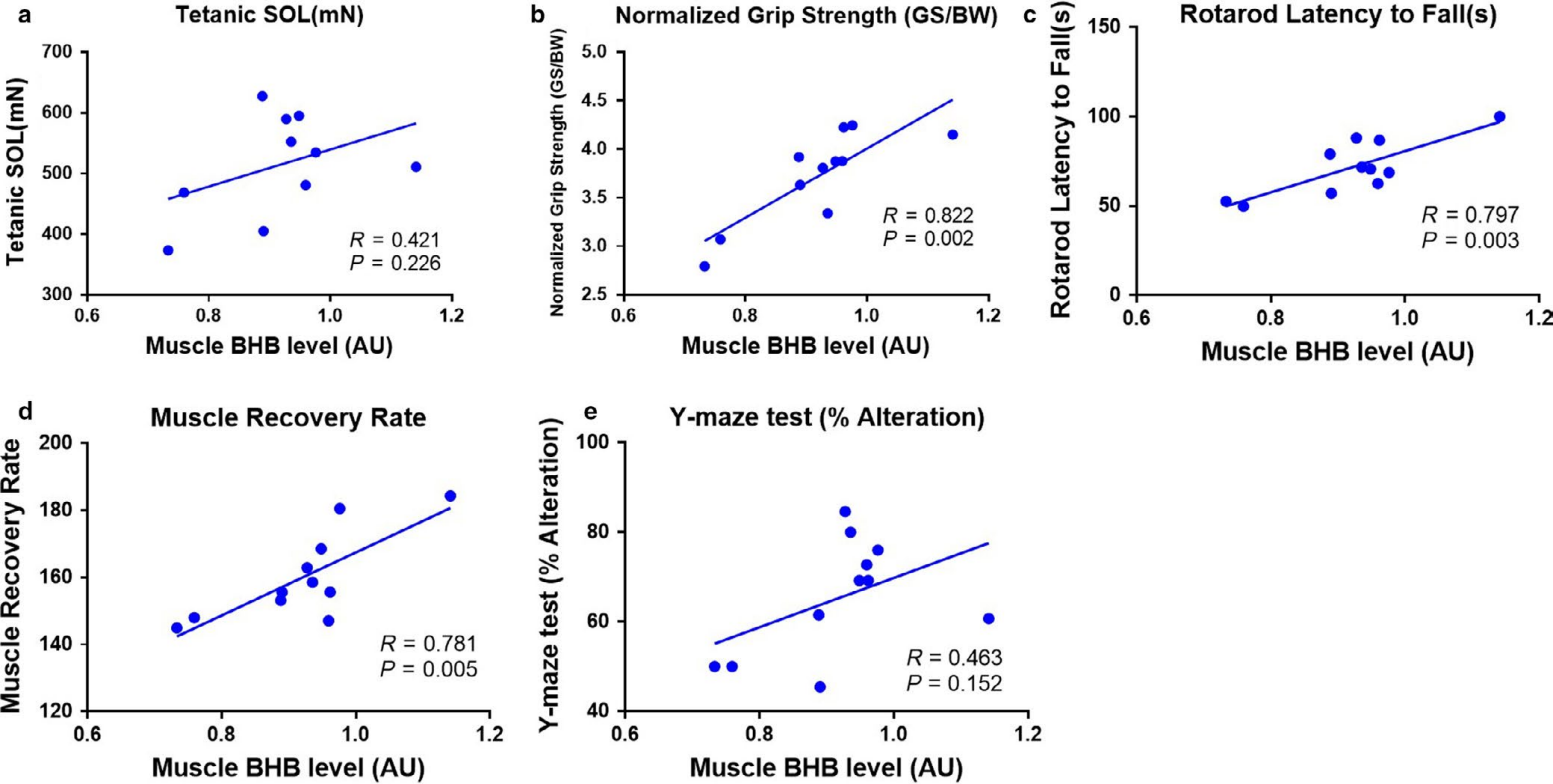
Relationships between beta‐hydroxybutyrate (BHB) and Muscle and Cognitive Function in Mice. (a–f) Correlations between soleus BHB concentration and muscle and cognitive function (*N* = 11). The ORE group was excluded. Linear regression lines are shown and Pearson's correlation coefficients are quoted

Because both the type of exercise and aging could affect the release of mediators from skeletal muscle, we first measured the BHB concentrations in skeletal muscle and serum. To determine the BHB concentration in aged mice, 26‐month‐old mice performed resistance exercise on a ladder or endurance exercise on a treadmill for 4 weeks, after which BHB was measured in the serum and SOL. There was a tendency for aged mice to have a lower serum BHB concentration than young mice, and the OEE group tended to have a higher concentration than the OC group. However, there was a tendency for the ORE group to have a lower concentration than the OEE group (Figure 1a). SOL concentrations showed similar trends to serum concentrations. Therefore, it seems that aging may reduce BHB concentration and endurance exercise, but not resistance exercise, may increase it (Figure 2b). It is likely that BHB concentration is increased only by endurance exercise because it inhibits glycolysis and activates the TCA cycle, which is consistent with the effects exercise previously shown in aged mice (Cahill & Aoki, 1980; Evans et al., 2017).

### 3.2 Relationships between BHB concentration and muscle and cognitive function in mice

Alteration percentage in Y‐maze test is an index of cognitive function in mice (Kwak, Cho, et al., 2019; Leinenga & Gotz, 2015; Park et al., 2019). In our previous study, alteration was downregulated by aging and upregulated by both types of exercise (Kwak, Cho, et al., 2019). Therefore, we analyzed the relationships between BHB concentration in skeletal muscle and cognitive and muscle function in mice, but excluded the resistance exercise groups, because they showed higher alteration but lower BHB concentration, because of its effects on glycolysis and the TCA cycle. Muscle BHB concentration positively correlated with isometric tetanic force (Figure 2a), grip strength (Figure 2b), rotarod latency to fall (Figure 2c), muscle recovery rate (Figure 2d), and Y‐maze alteration (%; Figure 2f). The skeletal muscle BHB concentration was significantly positively correlated with skeletal muscle function index, and cognitive function tended to have a positive relationship with BHB concentration in skeletal muscle.

### 3.3 Effect of BHB on skeletal muscle and glial cells

**FIGURE 3:**
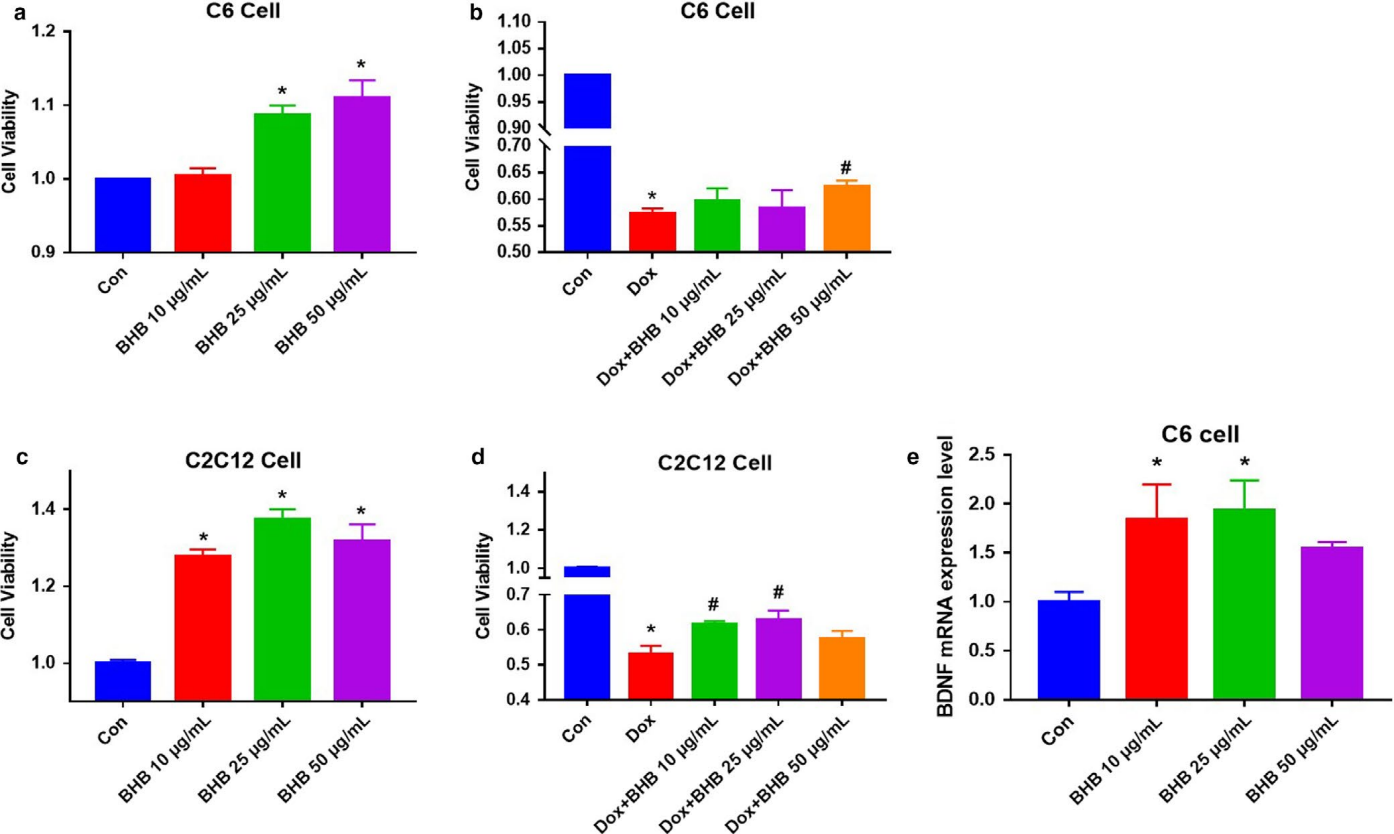
Effect of beta‐hydroxybutyrate (BHB) on Skeletal Muscle and Glial Cells. (a and b) Effects of BHB on skeletal muscle cells with and without doxorubicin (Dox) treatment. (c and d) Effects of BHB on glial cells with and without doxorubicin treatment. (e) Effects of BHB on C6 cells. *p < .05, versus Control. ^#^ *p* < .05, versus Dox. Statistical analysis was performed using one‐tailed and two‐tailed Student's *t* test. Data are means ± SEMs

Because there were relationships between skeletal muscle BHB and muscle and cognitive function (Figure 2a–e), we next determined whether there is a direct effect of BHB on skeletal muscle and glial cells in culture. Cell viability testing (Figure 3a–d) showed that BHB improved the viability of C2C12 and C6 glial cells (Figure 3a,c). In addition, we treated these cells with doxorubicin, which increases reactive oxygen species production (Kwak, Cho, et al., 2019), and found that BHB ameliorated its effects on cell viability in both cell types (Figure 3b,d). Furthermore, we measured the expression of a marker of cognitive function in the glial cells, BDNF (Pedersen, 2019), and found that BHB increased BDNF expression, which is consistent with an effect of BHB to improve cognitive function (Figure 3e). These results suggest that BHB has direct effects on skeletal muscle and glial cells and ameliorates the deleterious effects of doxorubicin.

### 3.4 The effect of BHB release from skeletal muscle and a BHB inhibitor on skeletal muscle and glial cells

**FIGURE 4:**
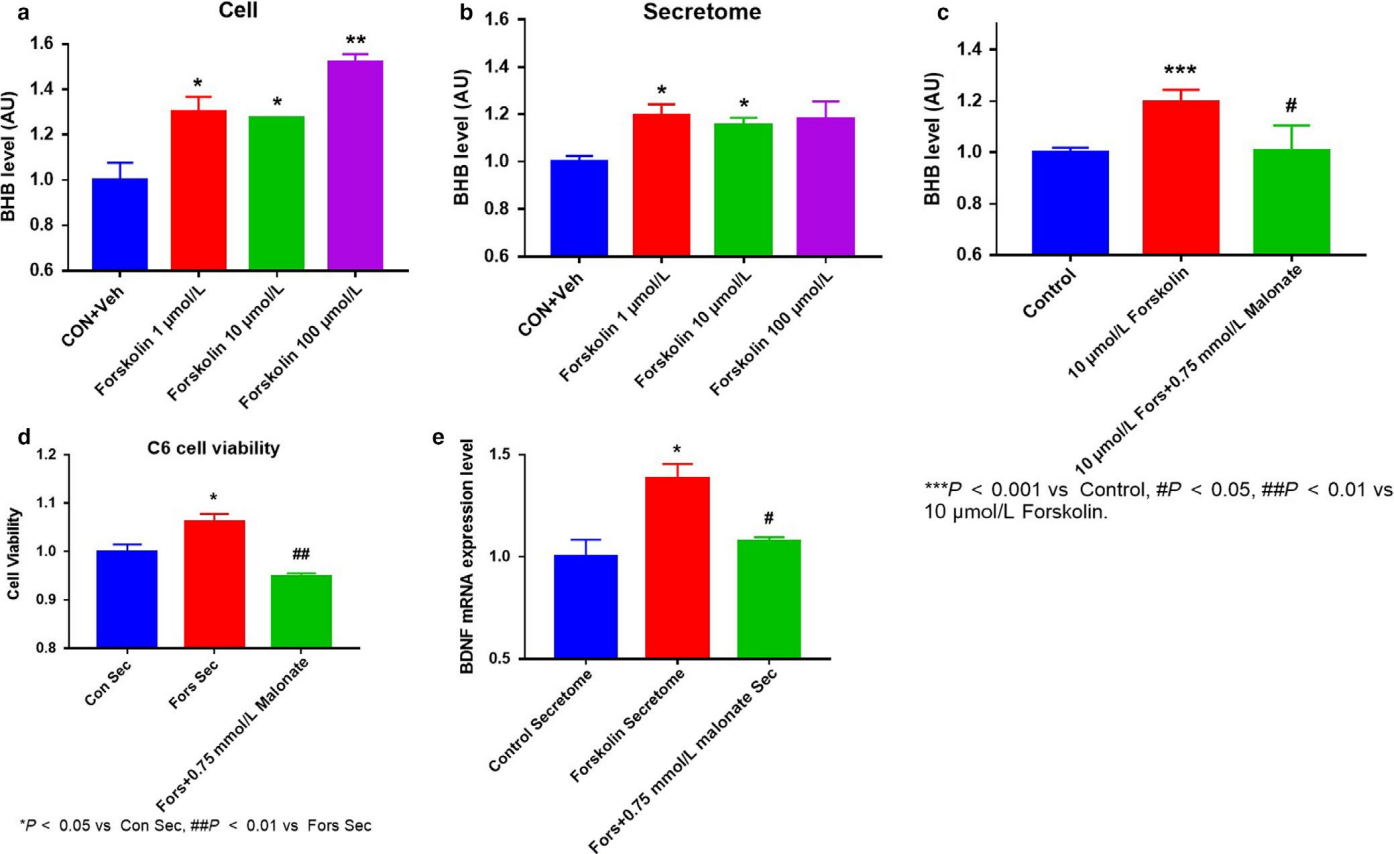
Effects of an exercise mimetic on skeletal muscle cell beta‐hydroxybutyrate (BHB) concentration and of muscle cell‐conditioned medium on glial cells. (a) Effect of forskolin on muscle cell BHB concentration. (b) Effect of forskolin treatment of muscle cells on the BHB concentration in the medium. (c) Effect of the addition of malonate during forskolin treatment. (d and e) Effects of conditioned medium on glial cell viability and BDNF expression. Statistical analysis was performed using two‐tailed Student's *t* tests. *p < .05, **p < .01, ***p < .001 versus Control, ^#^ *p* < .05, ^##^ *p* < .01 versus forskolin‐treated

To confirm the effects of BHB originating from exercising skeletal muscle, we treated cells with the exercise mimetic forskolin (Kwak, Cho, et al., 2019; Kwak, Shin, et al., 2019) and collected the skeletal muscle cells and the surrounding media. Both forskolin‐treated cell lysates and media contained higher BHB concentrations than control cells and media (Figure 4a,b), and this upregulation was prevented by the addition of malonate, an inhibitor of BHB production. We next treated C6 cells with muscle cell‐conditioned media, and found that the viability of the cells was higher in the forskolin‐treated muscle cell‐conditioned media and lower in malonate‐treated muscle cell‐conditioned media (Figure 4d). Furthermore, there were similar effects on BDNF mRNA expression (Figure 4e). These results suggest that exercise upregulates BHB release from skeletal muscle and that this BHB may improve the function of glial cells.

### 3.5 The effects of exercise on serum BHB concentration

**FIGURE 5:**
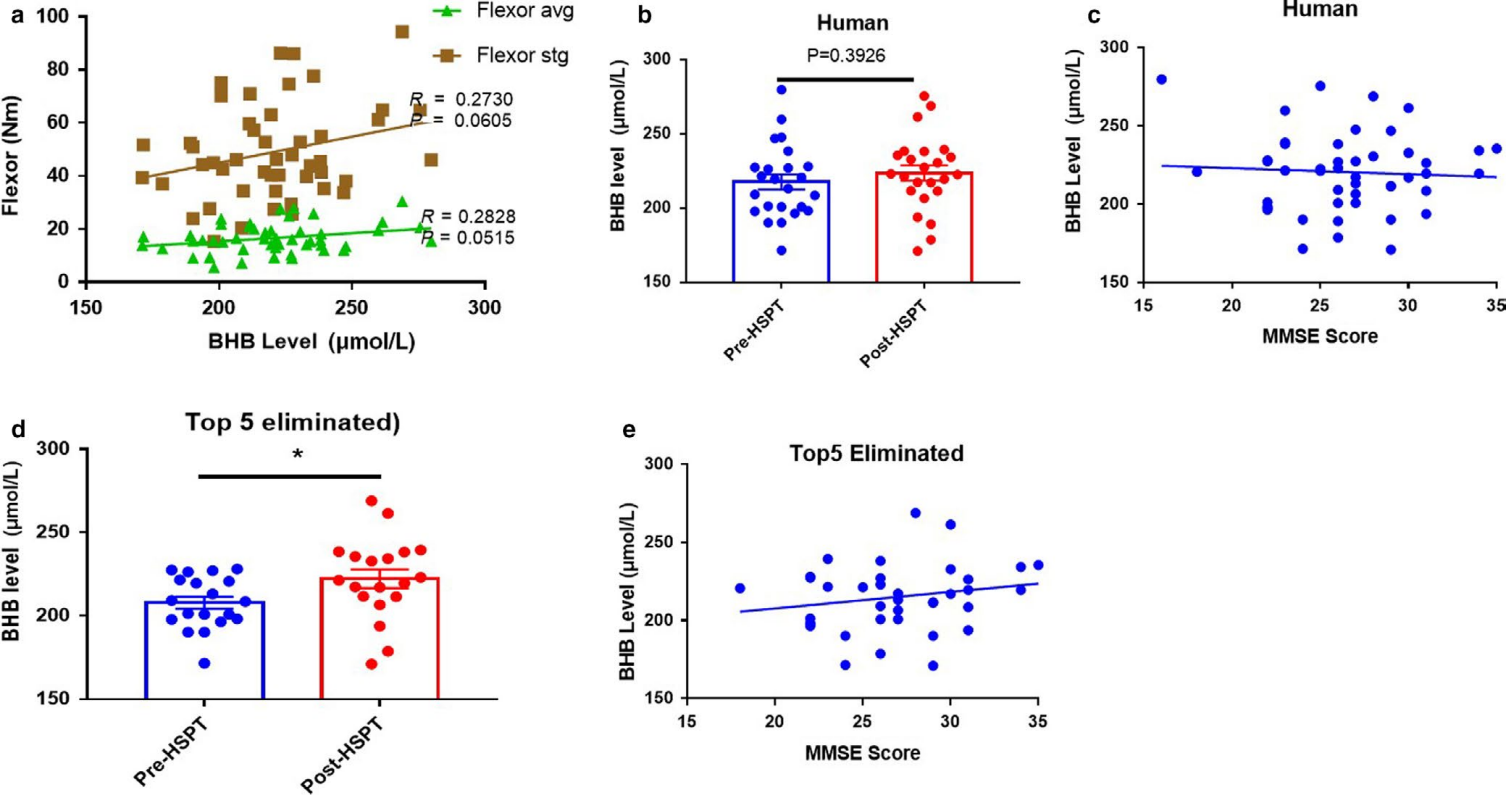
Effects of Exercise on Human Serum beta‐hydroxybutyrate (BHB) Concentration and the Relationships between BHB and Muscle and Cognitive Function. (a) Correlation between skeletal muscle function and BHB. (b) Effect of exercise on serum BHB concentration. (c) Correlation between cognitive function and BHB concentration. (d) Effect of exercise on serum BHB concentration after the exclusion of the five participants with the highest BHB concentrations. (e) Correlation between cognitive function and BHB concentration after the exclusion of the five participants with the highest BHB concentrations. *p < .05

**FIGURE 6:**
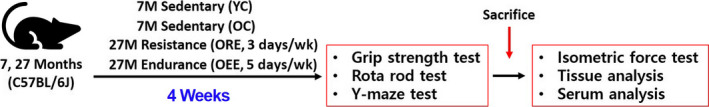
Experimental flow chart of the animal study

To determine the effects of exercise on BHB release and skeletal muscle and cognitive function, we measured the serum BHB concentration in a group of elderly women, who had performed 16 weeks of exercise, and have been described previously (Yoon et al., 2018). In this group, skeletal muscle function and BHB concentration were positively correlated; therefore, BHB may have a positive effect on skeletal muscle function (Figure 5a). However, no effects of exercise treatment and no correlation with cognitive function were apparent (Figure 5b,c). We speculated that individuals who already had high BHB concentrations might not affected by exercise; therefore, we excluded the five participants with the highest BHB concentrations, most of whom also had good skeletal muscle function (Figure 5a), and reanalyzed the data. In the remaining individuals, exercise was associated with high BHB concentration and there was a tendency for this to be associated with cognitive function (Figure 5d,e). Therefore, BHB released during exercise may improve skeletal muscle and cognitive function in elderly people (Figure 6).

## 4 DISCUSSION

We performed this study to determine the effects of BHB on muscle and cognitive function. We found that mimicking exercise in skeletal muscle cells increased BHB release and that conditioned medium had beneficial effects on glial cells. In an animal model, we have shown that BHB release is increased by endurance exercise and that BHB concentration positively correlates with skeletal muscle function, and to a lesser extent with cognitive function. Finally, in elderly women, we have shown that exercise increases serum BHB concentration and that there may be a positive relationship between BHB and cognitive and skeletal muscle function (Figure 5a–e). However, these results were obtained after excluding five individuals who had high BHB concentrations before exercise. Therefore, it may be that the effects of exercise‐induced BHB on cognitive function are only apparent in elderly people with lower BHB concentrations.

We found that BHB release was reduced by aging and increased by endurance exercise (Figure 1a,b), but resistance exercise did not increase BHB concentration in aged mice (Figure 1b). A previous study showed that BHB is used in the TCA cycle (Cahill & Aoki, 1980; Evans et al., 2017), which is activated by endurance exercise and is associated with substantial oxygen consumption (Howarth, LeBlanc, Heigenhauser, & Gibala, 2004). Conversely, activation of the glycolytic pathway would inhibit the TCA cycle; therefore, resistance exercise, which more heavily relies on glycolysis for its energy requirements, is likely to have less of an effect on BHB concentration (Egan & Zierath, 2013; LeBrasseur, Walsh, & Arany, 2011). In addition, a previous study showed that the positive effects of exercise on cognitive function were mostly associated with aerobic exercise. In contrast, effects of resistance exercise on cognitive function have rarely been shown; however, a meta‐analysis did show that resistance exercise has a positive effect on depressive symptoms (Gordon et al., 2018).

To compare resistance exercise and endurance exercise effect on BHB level and cognitive function in this animal study, we should have to equalize the amount of exercise in both groups, however, due to the age of the old mice group, 5 days/week for endurance exercise was appropriate for the old mice group, but 5 days/week for resistance exercise was too high intensity for the old mice group. During the study, the resistance exercise group died of aging and high load of resistance exercise, even though it was 3 days/week. Therefore, we could not equalize the amount of exercise duration and frequency. If the amount of exercise was equalized for both exercise groups, then its cognitive function improvement in resistance exercise group might be higher than our study; however, this result might be caused from other molecules that could effect on cognitive function, because resistance exercise uses BHB for energy metabolism, so resistance exercise does not upregulate BHB level (Cahill & Aoki, 1980; Evans et al., 2017).

We analyzed the relationships between BHB concentration and cognitive and skeletal muscle function in mice (Figure 2a–f), and found a positive relationship between BHB and skeletal muscle function. There may also be a positive relationship with cognitive function, but the correlation obtained was weak in the present study (Figure 2f). This may be because other molecules, such as irisin, BDNF, cathepsin B, and apelin, which are also released by exercising muscles, may have a greater effect on cognitive function (Moon et al., 2016; Pedersen, 2019).

The exercise intervention conducted in elderly volunteers did not affect serum BHB concentration and there was no correlation between BHB and cognitive function (Figure 5b,c). However, after the exclusion of the five individuals with the highest serum BHB concentrations before exercise, there was a significant increase in serum BHB following exercise in the remaining participants (Figure 5d). Furthermore, there was a positive relationship between cognitive function and BHB, although this did not reach significance (Figure 5e), and exercise‐induced BHB concentrations did not appear to have a substantial effect on cognitive function. This is likely to be because there are many exercise‐associated factors that affect cognitive function, such as BDNF, cathepsin B, irisin, and apelin (Islam et al., 2017; Kwak, Cho, et al., 2019; Moon et al., 2016; Pedersen, 2019). In addition, the exercise protocol was a hybrid form of exercise, which might not have had as big an effect on BHB as endurance exercise (Chan et al., 2016; Lee, Lee, & Kim, 2015). The individuals with preexisting high BHB concentrations may not have demonstrated increases in BHB that were as large as those in individuals with initially lower BHB concentrations, and in the present study, most of these participants already had good skeletal muscle function. Moreover, the effects of exercise type on cognitive function in the elderly should be considered, because most of the older individuals have sarcopenia, which has negative effects on exercise performance and skeletal muscle function, and is often associated with other chronic diseases. Future studies should determine which types of exercise would be more beneficial for both skeletal muscle and cognitive function in elderly people (Kim et al., 2019). A recent meta‐analysis of randomized control trials (RCTs) showed that aerobic exercise of low‐to‐moderate intensity improves cognitive function (Zheng, Xia, Zhou, Tao, & Chen, 2016). Thus, exercise type should be considered in determining the best method of preserving or improving cognitive function in the elderly.

Furthermore, BHB is kind of ketone body (Dedkova & Blatter, 2014; Robinson & Williamson, 1980), so it could be affected by daily diet; however, we could not control the diet of the participants. Therefore, it could affect the blood BHB levels in participants. Even though diet could have an effect on BHB level, it could be produced when starving situation is continued. By dieting, BHB could be produced when gluconeogenesis is curtailed or during glucose scarcity (Hashim & VanItallie, 2014). Therefore, although we did not control the diet of participants, unless participants stop the eating for a while, BHB level changing by diet would be insignificant.

In this study, BHB levels in serum and skeletal muscle were downregulated by aging. Previous studies also showed that BHB affects skeletal muscle function deficit and even cognitive function deficit such as Alzheimer's disease. However, BHB did not affect young models (Munroe et al., 2017; Pinto et al., 2018) therefore, it could be said that BHB is effective in elderly for muscle function and cognitive function.

It is difficult to identify molecules that are upregulated by exercise and have effects on cognitive function, because although effects can readily be observed in cells, there are numerous other molecules and signaling pathways that have effects on a physiological level. However, exercise is known to be one of the most effective ways of preventing deterioration in cognitive function (Almeida, Gomes da Silva, & Marques, 2019; Bae, Kwak, Lee, Yangjie, & Song, 2019; Di Liegro et al., 2019; Pedersen, 2019); therefore, the identification of molecules that link exercise with improvements in cognitive function in the elderly is important. Our findings that exercise‐induced BHB has beneficial effects on skeletal muscle and cognitive function may help determine the type of exercise that is most effective in improving skeletal muscle and cognitive function.

## CONFLICT OF INTEREST

The authors declare that the study was conducted in the absence of any commercial or financial relationships that could be construed as a potential conflict of interest.
